# Regulation of Swine Growth by Backfat Tissue during Growing and Finishing Stages

**DOI:** 10.3390/ani11123511

**Published:** 2021-12-09

**Authors:** Young-Jun Seo, Byeonghwi Lim, Do-Young Kim, Kyu-Sang Lim, Jun-Mo Kim

**Affiliations:** 1 Department of Animal Science and Technology, Chung-Ang University, Anseong 17546, Gyeonggi-do, Korea; tjdudwns201@cau.ac.kr (Y.-J.S.); hwi1208@cau.ac.kr (B.L.); dodamx2@cau.ac.kr (D.-Y.K.); 2 Department of Animal Science, Iowa State University, Ames, IA 50011, USA; kyusang0912@gmail.com

**Keywords:** backfat, KNP, pig, RNA-seq, transcriptome

## Abstract

**Simple Summary:**

Swine have a large influence on livestock animals. In particular, Korean native pigs (KNPs) have unique traits in their body composition including lipids and proteins. In this study, we performed RNA-sequencing analysis to identify porcine transcriptomic changes during growing and finishing stages in the backfat tissue of KNP and Yorkshire pig crossbreeds. Enrichment analysis revealed that differentially expressed genes (DEGs) were significantly influenced by lipid metabolism and hormonal changes. Network analysis showed that the *LEP* and *ACTC1* genes were connected with significant terminologies which resulted from up- and down-regulated DEGs. The results of our analysis indicate that backfat tissue could regulate swine biology during stages of growth. Consequently, our analysis provided comprehensive understanding for transcriptomic changes during growth periods.

**Abstract:**

Recently, interest in the function of pig backfat (BF) has increased in the field of livestock animals, and many transcriptome-based studies using commercial pig breeds have been conducted. However, there is a lack of comprehensive studies regarding the biological mechanisms of Korean native pigs (KNPs) and Yorkshire pig crossbreeds. In this study, therefore, BF samples of F1 crossbreeds of KNPs and Yorkshire pigs were investigated to identify differentially expressed genes (DEGs) and their related terms using RNA-sequencing analysis. DEG analysis identified 611 DEGs, of which 182 were up-regulated and 429 were down-regulated. Lipid metabolism was identified in the up-regulated genes, whereas growth and maturation-related terminologies were identified in the down-regulated genes. *LEP* and *ACTC1* were identified as highly connected core genes during functional gene network analysis. Fat tissue was observed to affect lipid metabolism and organ development due to hormonal changes driven by transcriptional alteration. This study provides a comprehensive understanding of BF contribution to crossbreeds of KNPs and Yorkshire pigs during growth periods.

## 1. Introduction

Pigs are important animals that fulfil a significant proportion of global meat demand [1,2]. Fat deposition and growth rates are significant economic traits in swine and have been intensively studied in pig genetics [3]. In particular, fat deposition has been shown to strongly affect production efficiency, meat quality, and reproduction performance [4,5]. Adipose tissue has been reported to have functions related to fatty acid synthesis, fat and energy storage, fat deposition, and metabolism regulation in pigs. Backfat (BF), which is a type of adipocyte, can directly indicate the deposition of pig fat among morphological traits. Indeed, BF thickness is currently used as a valuable indicator to monitor productivity because of its negative correlation with lean meat production ability [6,7]. Recent studies have shown that BF affects traits relating to important factors including meat quality, reproduction, and growth rate [8,9]. Additionally, genetic analysis of BF has revealed quantitative trait loci (QTL) associated with *ADIPOQ*, *LEPR*, *ELOVL6*, and *FAT1* genes in wild boar *Sus scrofa* chromosomes (SSC) 2, SSC 6, SSC 8, SSC 13, and SSC 17 to identify genes that contribute to metabolism in pigs [10,11,12,13,14].

Notably, adipose tissues, including BF, have been extensively reported to secrete adiponectin, leptin, resistin, etc., which are associated with skeletal muscle growth, cellular growth, obesity, diabetes, and metabolic diseases [15,16]. These proteins have previously been reported to be related to growth hormones using next-generation sequencing [17]. Proteins that are known to be secreted by adipose tissues are notably related to growth [13,18]. Moreover, dynamic changes in number and size of adipose tissue over 18–20 weeks are reported in swine [19]. That is, previous research suggests that there could be a large variation in BF tissue between the beginning and end periods of the growing and finishing stages. However, there have been few transcriptomic-based studies into the growth effects of adipose tissue. Nonetheless, elucidating the pig transcriptome is essential for interpreting the functional prediction of the genome and also for understanding complex traits such as growth and fat deposition.

The Korean native pig (KNP) is a breed of pig indigenous to the Republic of Korea [20]. It has been reported to contain higher levels of unsaturated fatty acid and polyunsaturated fatty acid in its muscle tissue than those of other breeds, but it has a lower growth rate and poorer reproductivity [21,22]. Because of these characteristics, the need for crossbred pigs that offset the disadvantages of KNPs through crossbreeding with commercial pigs has emerged. In this circumstance, the Yorkshire pig has been selected to crossbreed with KNP due to high growth performance and reproductivity compared with KNPs [23]. F1 individuals produced through crossbreeding take advantage of non-additive gene effects and produce offspring of higher quality than both parents. However, the crossbred animals’ biological traits differ from purebred animals, and further research is needed to uncover useful heterosis when they are crossed. Recently, gene expression patterns of animals were considered as an intermediator between genotypes and phenotypes of heterosis [24,25]. For this reason, useful heterosis selection can be achieved by a holistic understanding of the gene expression patterns in heterosis. Although research about QTL or lncRNA has been continuously conducted for the F1 breed of the hybrid, there is still a lack of research on the BF of the hybrid F1 breed using RNA-sequencing, which would allow an understanding of gene expression patterns in a comprehensive way. Therefore, the aim of this study is to decode the biology of the KNP and Yorkshire pig crossbreed F1 by confirming the changes in the BF tissue during the growth period at the transcriptome level based on functional enrichment analysis.

## 2. Materials and Methods

### 2.1. Experimental Animals and Sample Collection

In this study, 12 F1 crossbreeds of KNPs and Yorkshire pigs were used for analysis. This study was conducted with approval from the Institutional Animal Care and Use Committee (IACUC; no. NIAS2016-848). The 12 pigs were raised and equally divided into groups of 10 weeks of age (10 W) and 26 weeks of age (26 W), corresponding to the beginning (growing) and end (finishing) periods of the growing and finishing stages, respectively. Both groups were fostered by the self-serving of feed under standard environmental conditions. At the end of each growing period (at 10 W and 26 W), the animals were slaughtered and BF tissues were collected for RNA extraction. The BF tissue was dissected into pieces (1 cm^3^), rapidly frozen using liquid nitrogen, and then stored at −80 °C.

### 2.2. RNA Extraction, Library Preparation, and Sequencing

TRIzol reagent (Invitrogen, Carlsbad, CA, USA) was used to extract the total RNA for each sample. Quality-checked RNA (NanoDrop, Thermo Scientific, Waltham, MA, USA) was used to synthesize complementary deoxyribonucleic acid (cDNA), and the cDNA samples underwent 5′ and 3′ adapter ligation before being randomly fragmented. During flow cell analysis, fragments were caught on a lawn of surface-bound oligos complementary to the library adapters for cluster generation. Then, each fragment was amplified into clonal, distinct clusters using bridge amplification. After the library was loaded into a flow cell, polymerase chain reaction (PCR) amplification and gel purification were conducted for the adapter-ligated fragments. Messenger RNA sequencing was performed using an Illumina HiSeq 4000 (Illumina Inc., San Diego, CA, USA) sequencer using paired-end, 100 bp reads.

### 2.3. DEG Profiling

The quality of the raw read data was checked using FastQC (Babraham Bioinformatics; http://www.bioinformatics.babraham.ac.uk/projects/fastqc/, accessed on 20 September 2020). The sequences of adapters and low quality reads were removed using Trimmomatic v.0.38 [26] to isolate clean reads. The reference genome of *Sus scrofa* (v.11.1) was used in the FASTA format from Ensemble (http://www.ensembl.org/, accessed on 20 September 2020). Clean, paired-end reads were mapped against an indexed reference genome using HISAT2 [27], which is a sensitive and fast alignment program for next-generation sequencing reads. The raw counts corresponding to the genes for each library were calculated based on exons and the Sus_scrofa v98 GTF file was taken as the genomic annotation reference file using the feature count function of the R package ‘Subread’ [28].

Raw counts were normalized using the trimmed means of M values (TMM) method [29] in the R package ‘edgeR’ [30], following which DEG profiling was conducted by comparing the normalized read counts between the 10 W and 26 W groups. The false discovery rate (FDR) was calculated using the Benjamin–Hochberg procedure. Significant DEGs were extracted by applying the thresholds of FDR < 0.05 and absolute log_2_ fold change (FC) ≥1. The overall expressions of the genes were visualized using the R package ‘ggplot’.

### 2.4. Functional Enrichment Analyses

To investigate the functional enrichment analyses of DEGs, the database for annotation, visualization, and integrated discovery (DAVID) bioinformatics resources 6.8 was used to assess the Kyoto encyclopedia of genes and genomes (KEGG, https://www.genome.jp/kegg/, accessed on 20 September 2020) and gene ontology (GO, http://geneontology.org/, accessed on 20 September 2020) databases. In this analysis, the significances of KEGG and GO in each stage were detected at *p* < 0.1. The results of the KEGG and GO analyses were visualized by using −log_10_ *p* and log_2_ fold enrichment as criteria. GO enrichment analysis was conducted in three categories: biological process (BP), cellular component (CC), and molecular function (MF). The enriched GO terms were visualized as tree maps using the REVIGO [31] visualization tool. The networks of gene clusters in functional groups were also visualized using the ClueGo and CluePedia plugins (v.2.5.5) of Cytoscape (v.3.7.2) [32].

The expression data for gene set enrichment analysis (GSEA) [33] were collated using the TMM-normalized count data, and all genes were ranked within a dataset based on their expressional differences to assess the expression patterns between the two groups. The GO database was used to further process the gene set data, and the parameters were set at their default values. Then, gene sets were ranked by calculating the normalized enrichment scores (NESs). After GSEA, the results were visualized using a functional enrichment map in Cytoscape (v.3.7.2) [34]. The cut-off value was FDR < 0.01. The similarity cut-off was an overlap coefficient value of >0.8. In addition, the top 50 terms of the GSEA results were visualized using the NESs and gene set size as criteria.

## 3. Results

### 3.1. DEG Profiling

The RNA-seq data processing results showed that the 12 total samples between the 10 W and 26 W groups had an average of 34,923,354 raw reads and an average guanine–cytosine (GC) content of 50.82%. After trimming, the average number of reads was 34,628,356, and the GC content was 50.80%. The mapping results presented that 70.99% of the reads were uniquely mapped and the overall mapped rate was 92.87% (Table 1).

**Figure 1:**
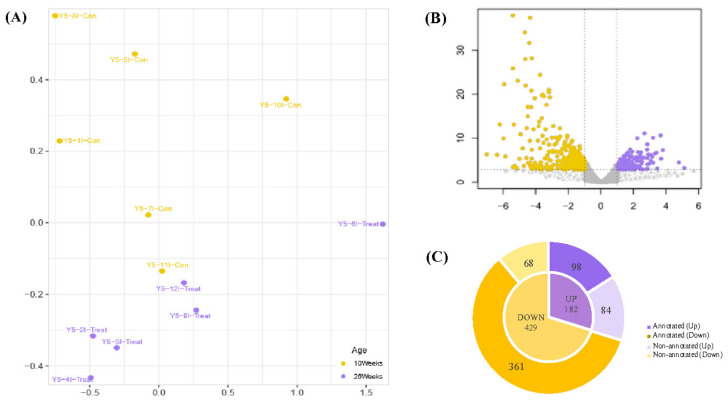
RNA-seq transcriptional profiling in swine backfat (BF). (**A**) Multidimensional scaling (MDS). Each point represents an individual sample. The yellow and purple dots represent control (10 W) and treatment (26 W) samples. (**B**) Volcano plot showing overviews of differentially expressed genes (DEGs). The log_2_ fold changes (FCs) in gene expressions for the 26 W:10 W ratio and −log_10_ *p* are shown on the x and y axes, respectively. The purple and yellow dots represent significantly up- and down-regulated DEGs, respectively (false detection rate [FDR] < 0.05, absolute log_2_ FC ≥ 1). Gray dots indicate non-significant DEGs, the horizontal line indicates the significance threshold (FDR < 0.05), and the vertical lines indicate the two FC thresholds (absolute log_2_ FC ≥ 1). (**C**) Each annotated and non-annotated up- and down-regulated DEG. Light and dark gray sections show annotated and non-annotated genes, respectively.

The multidimensional scaling (MDS) plot indicated transcriptome-based similarities among the samples of the 10 W and 26 W groups (Figure 1A), showing that dots were distinctively clustered into each group. Volcano plots show the thresholds and the DEGs between the two groups (Figure 1B). A total of 611 DEGs were identified, including 182 up-regulated (high expression in 26 W) and 429 down-regulated (low expression in 26 W) genes (Table S1). Among the up-regulated genes, 98 (53.85%) were annotated, while 361 (84.15%) of the down-regulated genes were annotated (Figure 1C).

### 3.2. Functional Annotations

**Figure 2:**
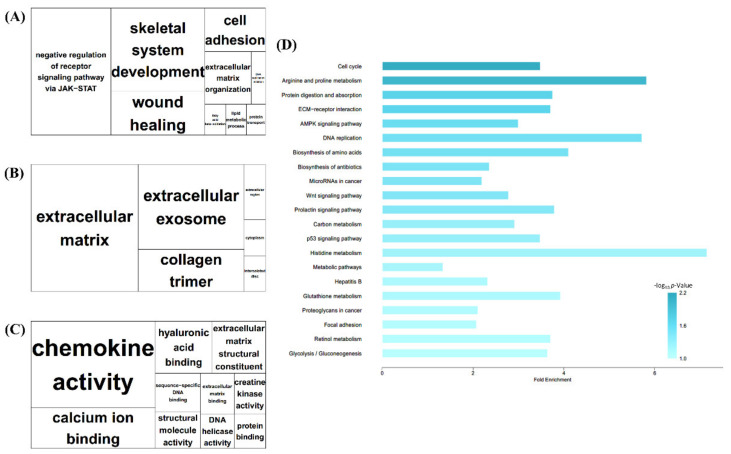
Gene Ontology (GO) tree map and Kyoto Encyclopedia of Genes and Genomes (KEGG) pathways of Total DEGs. (**A**–**C**) tree map views of GO analysis of BF tissue. Box size represents −log_10_ *p* of GO term enrichment, as indicated by bold letters. (**A**) Biological process (BP). (**B**) Cellular component. (**C**) Molecular Function (**D**) KEGG pathway of total DEGs. X axis indicates fold enrichment value and darker-colored bars indicate higher −log_10_ *p*.

The significantly enriched GO terms in BP, CC, and MF are shown in Figure 2A–C. BP indicated terms related to cell and protein signaling, such as skeletal system development, regulation of cell growth, and protein transport. It also indicated terms related to inflammatory functions, such as wound healing. In CC, extracellular matrix terms, including proteinaceous terms, were the largest, whereas, in MF, chemokine activity was the largest term.

The significant pathways were identified based on the KEGG database (Figure 2D). The KEGG results were similar to the GO results regarding the identified growth and protein-metabolism-related terms. In addition, the adenosine monophosphate-activated protein kinase (AMPK) signaling pathway, which can be related to lipolysis and the immune-associated biosynthesis of antibiotics, was also enriched with respect to the total DEGs.

GO analysis was also conducted using up- and down-regulated DEGs. First, in the up-regulated DEGs (Figure S1A–C), ‘Brown fat differentiation’ notably appeared in the BP category (Figure S1A). ‘Oxidoreductase activity’ and ‘Heme binding’ were also notable in the MF category regarding up-regulated DEGs (Figure S1C). In down-regulated DEGs, ‘Anatomical structure morphogenesis’ and ‘Cell adhesion’ appeared in the BP category (Figure S1D), ‘Extracellular matrix’ and ‘Proteinaceous extracellular matrix’ were revealed in the CC category (Figure S1D), and ‘Calcium ion binding’ and ‘Structural molecular activity’ appeared in the MF category (Figure S1D).

**Figure 3:**
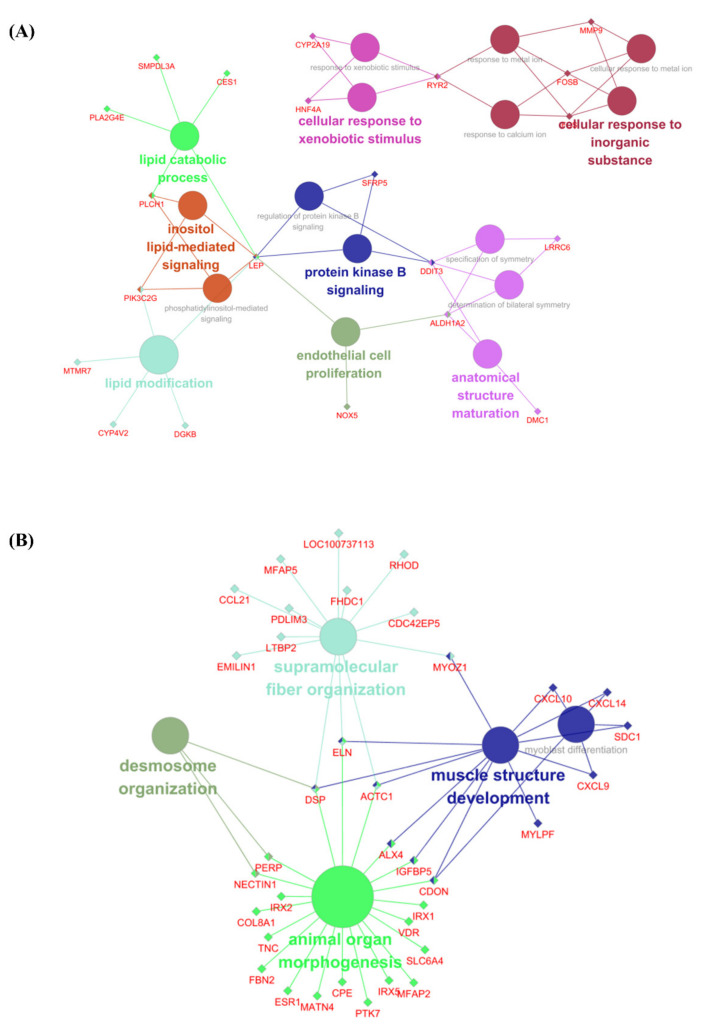
The networks of gene clusters in functional groups were chosen for BP network by ClueGo + CluePedia. Significant DEGs are shown (FDR < 0.05 and absolute log_2_ FC ≥ 1.0). Parameters of ClueGo: min level = 3, max level = 8, and 3 min# genes = 2.000% genes. The represented pathway is *p* < 0.05. The related BP terms are the same color and notable genes are clustered. (**A**) The networks of gene clusters in a functional group using significant up-regulated DEGs. (**B**) The networks of gene clusters in a functional group using significant down-regulated DEGs.

Figure 3A,B indicate networks based on the GO terms for up-regulated and down-regulated DEGs, respectively. The up-regulated DEG network reveals that they were involved in 16 detected pathways and 21 genes. Terms that related to lipid metabolism (‘Lipid modification’, ‘Lipid catabolic process’, ‘Protein kinase B signaling’, and ‘Inositol-mediated signaling’) were identified. These nodes were connected with one gene, Leptin (*LEP*). Terms related to anatomical structure maturation and endothelial cell proliferation were also revealed. The network of down-regulated DEGs consists of 5 nodes and 36 genes (Figure 3B). The detected nodes were generally related to growth processes such as muscle structure development, animal organ morphogenesis, and supramolecular fiber organization. The genes *ELN*, *DSP*, and *ACTC1* were found to be connected to more than three terms each in the network.

Regarding significant (*p* < 0.01) KEGG pathways for up- and down-regulated DEGs, ‘Retinol metabolism’ and ‘Histidine metabolism’were identified regarding up-regulated DEGs (Figure S2A, Table S3), while pathways related to pig growth terms were identified regarding the down-related DEGs, such as ‘Cell cycle pathway’ and ‘Protein differentiation and absorption’ (Figure S2B).

**Figure 4:**
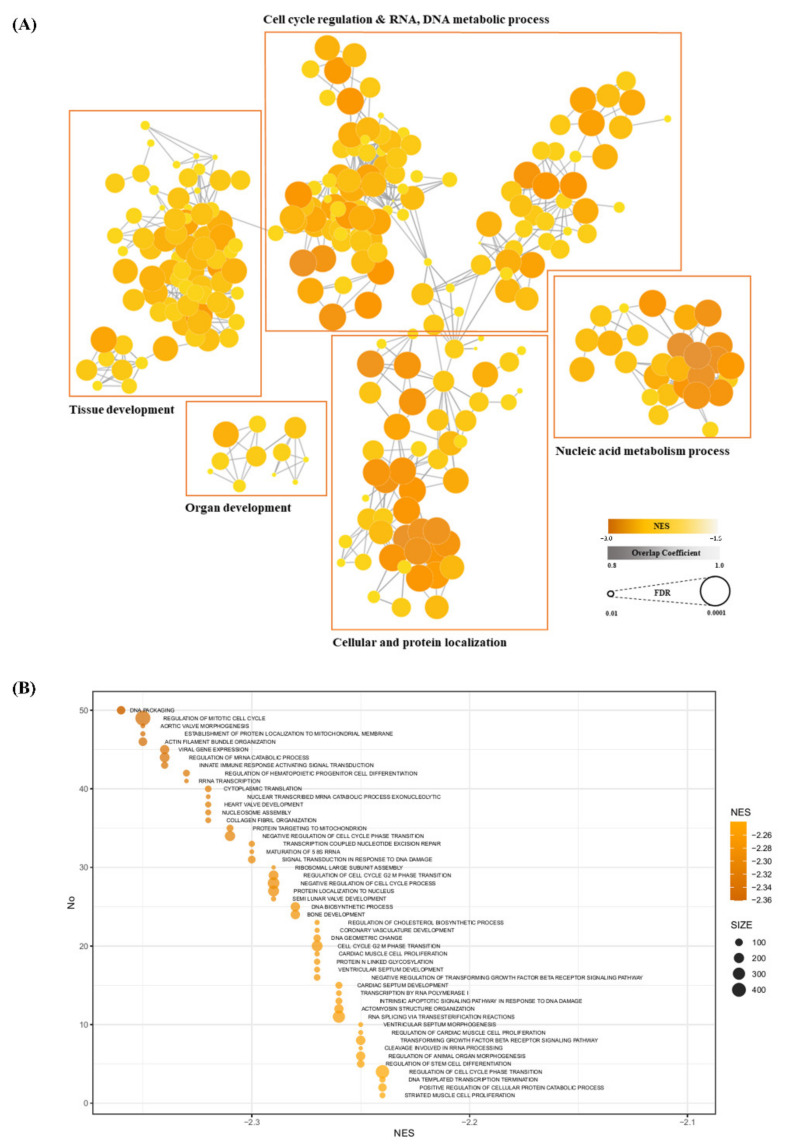
Gene set enrichment analysis (GSEA) for BP. (**A**) Cytoscape visualizing functional enrichment analysis results from GSEA. Nodes represent each enriched BP term from GO. Thicknesses of lines represent overlap coefficients (levels of overlap between nodes). Node color and size indicate normalized enrichment scores (NESs) and false discovery rate (FDR) each. Nodes are clustered into groups of five by similarity of each function. (**B**) Top 50 terms regarding NESs in GSEA, shown as a bubble plot. Each node contains an NES and a gene set size value. Terms are involved in nucleic acid metabolism, cell cycle regulation, and cellular and protein localization.

Enrichment scores (ESs) and estimated significance levels of ESs in each group were calculated, with the GSEA results being visualized as a network based on the significant terms. Clusters were formed by combining representative functions (Figure 4A). In this network, eight clusters were identified by node functional similarity; details of each cluster’s composition are given in Table S4. Among them, clusters related to organ and muscle development showed significantly higher absolute NESs. The colors of each node relate to each NES. This network is composed of similarity coefficients between the terms of GSEA results, with each term being expressed in terms of its NES and FDR significance. The top 50 terms of the GSEA results are shown in Figure 4B, indicating that ‘DNA packaging’, and ‘Regulation of Miotic Cell cycle’, which are associated with cell cycles, had the highest NESs.

## 4. Discussion

In this study, transcriptome profiles in the BF tissue of KNP crossbreeds were investigated regarding differences between the 10 W and 26 W stages, which is the time when BF tissue changes dynamically. In total, 611 DEGs were identified between the two groups, including 182 up-regulated and 429 down-regulated DEGs. Among them, 98 and 361 were annotated for up- and down-regulated DEGs, respectively. Subsequently, functional annotations and network analyses were conducted for these DEGs to identify the significant mechanisms of each stage.

Regarding the functional analyses of up-regulated DEGs in BF tissue, terms notably related to lipid metabolism (lipid catabolic process and lipid modification) are shown in Figure 3. A previous study reported that pigs reached their highest fat content (~15%) at 21 days after birth [35]; thus, the fat synthesis gene becomes progressively less expressed after 21 days of postnatal development [35]. Similarly, here ‘Lipid catabolic process’ and ‘Protein kinase B signaling’, which include the degradation of fat through the Akt pathway [36], were significant in the 10 W group. Therefore, this indicated a decrease in fat synthesis depending on the growth rate, corresponding to the previous studies [35,36].

During growth, Somatotropin (pST), which is a peptide hormone, is steadily produced in the pituitary glands of animals. pST increases the deposition of lean muscle tissue and decreases fat accumulation in growing pigs [37]. The mechanism by which pST achieves nutrient partitioning comprises the modulation of tissue responsiveness to insulin. Reducing insulin sensitivity decreases insulin-regulated mechanisms (lipogenic enzyme activities, glucose transport, and lipogenesis). These mechanisms can reduce fatty acid synthesis in adipose tissue during the growing stage [38,39,40]. According to these findings, it can be inferred that up-regulated DEGs in the present study were affected by growth hormone changes involved in lipid synthesis between the two stages (10 W and 26 W).

The most notable up-regulated DEG identified during DEG profiling and network analysis in BF tissue was *LEP*. In our results, *LEP* contributes to lipid metabolism and insulin secretion in functional analysis with the GO database (Table S2). Moreover, *LEP* was also shown as a core gene that connects main pathways such as lipid catabolic process, inositol lipid-mediated signaling, and protein kinase B signaling in network analysis (Figure 4A). *LEP* has been associated with adipose tissue causing polymorphisms and regulating porcine hormones [41,42,43]. In addition, *LEP* has been found to prevent obesity by contributing to lipolysis metabolism [44]. In agreement with the results of this previous study, here *LEP* was considered to be involved in lipid modification and lipid catabolic processes. In addition, *LEP* has been reported to be an important regulator of appetite control and energy metabolism as a neuromodulator [44]. This reported *LEP* function was similarly shown here as an ‘Eating behavior’ term involved with *LEP* (Figure S1A). Protein kinase B is known to play an important role in insulin metabolism [36]. *LEP* has also been reported to affect insulin-related metabolism by regulating the expression and secretion of multiple neurotransmitters, neuropeptides, and hypothalamic hormones, including neuropeptide-Y, galanin, melanin-concentrating hormone, gonadotropin-releasing hormone, corticotropin-releasing hormone, growth hormone-releasing hormone, somatostatin, and thyroid-releasing hormone [44]. This study confirmed that *LEP* had a large influence on the porcine lipid accumulation mechanism during early stages and that it promotes lipolysis metabolism, as previously studied. According to these results, *LEP* might play a significant role in lipid metabolism and lipolysis processes during growing periods.

The down-regulated DEGs identified in BF tissue showed similar results to the total DEGs identified in functional profiling. They were related to processes such as morphogenesis, organ development, and organization. However, terms related to maturity were mainly found in the 26 W group in up-regulated DEGs (identified via GO network analysis), including anatomic structure maturation. Therefore, the pigs may have transcriptomic changes related to muscle and organ growth after the post-weaning stage.

*ACTC1*, *DSP*, and *ELN* were identified at the centers of terms of down-regulated DEGs during networks analysis, but only *ACTC1* wasdifferenctially expressed between two groups. *ACTC1* encodes for a protein that is involved in the molecular functions of actin and in dysfunction during skeletal muscle development [45]. *ACTC1* also promotes the structural integrity of the cytoskeleton [46]. Moreover, *ACTC1* is associated with the developmental stage and has been reported to be more highly expressed in very young pigs before gradually lowering [47]. Therefore, the decrease in the development rate of newly generating tissues means that growth-related terms regarding muscles and organs appear along with the decrease in *ACTC1* expression.

Consequently, here DEG profiling and functional annotation results indicated that dynamic genomic changes occurred between the post-weaning and end of finishing periods. In addition, crossbreeds of KNPs and Yorkshire pigs, which were used in this study, have similar key genes to other commercial pigs in their BF tissues. As already reported, the results of the present study also indicate that BF tissue has a large effect on porcine hormones, organ morphology, and adipose tissue synthesis. Therefore, this study could be used to identify transcriptomic changes in BF tissue during the growing and finishing periods of pigs. Additionally, the observed changes indicate that BF tissue could influence lipid metabolism and organ development as a result of hormonal alterations (through transcriptional alterations). These observations suggest that crossbreeds of KNPs and Yorkshire pigs have overall similar biological traits of BF to other commercial breeds during growth and provide candidate genes to breed crossbreeds of KNPs and Yorkshire pigs in a more efficient way.

However, one limit of this study is that the time points were divided in detail, which complicated the determination of BF mechanisms in each growth period. Additionally, further studies will be needed to quantitatively validate the candidate genes identified in this study using quantitative real-time PCR. It is also necessary to validate the identified candidate genes using larger populations with more subdivided periods and to use an epigenetic approach to understand the effects of the environment on animal transcriptomics [48].

## 5. Conclusions

We conducted transcriptome profiling of F1 crossbreeds of KNPs and Yorkshire pigs in the beginning and end periods of growing and finishing. We then performed functional analysis of DEGs in each of the stages. Consistent with the results of previous studies, pathways and networks related to lipid metabolism, morphological function, and hormone change are largely represented in DEGs. Furthermore, the network analysis provided candidate genes (*LEP* and *ACTC1*), which could influence growth in crossbred KNPs and Yorkshire pigs. Our findings may contribute to a comprehensive understanding of BF tissue during the growth stage for crossbreeds of KNPs and Yorkshire pigs.

## Figures and Tables

**Table 1:** Summary of ribonucleic acid sequencing (RNA-seq) raw reads, trimming, and mapping of swine genome.

Group	Sample Name	Raw		After Trimming			After Mapping	
		Total	GC (%)	Remaining Reads after Trimming	Trimmed GC (%)	Trimming Rate (%)	Uniquely Mapped Rate (%)	Overall Mapped Rate (%)
10 weeks	Y5-1l	36,025,628	51	35,762,755	51	0.73	79.27	95.77
	Y5-5l	31,917,774	51	31,628,028	51	0.91	77.28	94.94
	Y5-7l	29,776,194	50	29,565,748	50	0.71	73.81	91.95
	Y5-8l	31,526,567	52	31,270,069	52	0.81	72.46	93.22
	Y5-10l	31,661,523	58	31,332,164	58	1.04	44.99	88.17
	Y5-11l	32,965,477	54	32,684,669	54	0.85	64.63	94.07
26 weeks	Y5-2l	35,158,741	48	34,906,703	48	0.72	82.01	95.30
	Y5-3l	37,475,105	48	37,264,232	48	0.56	78.73	94.05
	Y5-4l	49,961,237	51	49,411,205	51	1.10	65.19	94.86
	Y5-6l	29,944,115	48.49	29,593,083	48.49	1.17	63.47	84.46
	Y5-9l	33,926,962	47	33,652,163	47	0.81	78.71	93.10
	Y5-12l	38,740,925	49	38,469,448	49	0.70	71.37	94.58
Average		34,923,354	50.82	34,628,356	50.8	0.84	70.99	92.87

## Data Availability

Illumina sequencing raw reads data have been uploaded to the NCBISRA database, item number PRJNA768488 (https://www.ncbi.nlm.nih.gov/sra/PRJNA768488, accessed on 1 October 2021).

## Supplementary Materials

The following are available online at https://www.mdpi.com/article/10.3390/ani11123511/s1, Figure S1: Revigo GO term analysis, Figure S2: KEGG pathway graph, Table S1: DEGs list, Table S2: Gene ontology analysis, Table S3: Kegg pathway, Table S4: GSEA analysis.

## References

[1] Sans P., Combris P. (2015). World meat consumption patterns: An overview of the last fifty years (1961–2011). Meat Sci..

[2] Knecht D., Duziński K. (2016). The effect of sex, carcass mass, back fat thickness and lean meat content on pork ham and loin characteristics. Arch. Anim. Breed..

[3] Bosi P., Russo V. (2004). The production of the heavy pig for high quality processed products. Ital. J. Anim. Sci..

[4] Liu Y., Kong X., Jiang G., Deng J., Yang X., Li F., Xiong X., Yin Y. (2015). Effects of dietary protein/energy ratio on growth performance, carcass trait, meat quality, and plasma metabolites in pigs of different genotypes. J. Anim. Sci. Biotechnol..

[5] Ibáñez-Escriche N., Magallón E., Gonzalez E., Tejeda J., Noguera J. (2016). Genetic parameters and crossbreeding effects of fat deposition and fatty acid profiles in Iberian pig lines. J. Anim. Sci..

[6] Maes D., Janssens G., Delputte P., Lammertyn A., de Kruif A. (2004). Back fat measurements in sows from three commercial pig herds: Relationship with reproductive efficiency and correlation with visual body condition scores. Livest. Prod. Sci..

[7] Cho K., Kim M., Jeon G., Chung H. (2011). Association of genetic variants for FABP3 gene with back fat thickness and intramuscular fat content in pig. Mol. Biol. Rep..

[8] Suzuki K., Irie M., Kadowaki H., Shibata T., Kumagai M., Nishida A. (2005). Genetic parameter estimates of meat quality traits in Duroc pigs selected for average daily gain, longissimus muscle area, backfat thickness, and intramuscular fat content. J. Anim. Sci..

[9] Tummaruk P., Lundeheim N., Einarsson S., Dalin A.-M. (2001). Effect of birth litter size, birth parity number, growth rate, backfat thickness and age at first mating of gilts on their reproductive performance as sows. Anim. Reprod. Sci..

[10] Fontanesi L., Schiavo G., Galimberti G., Calò D.G., Scotti E., Martelli P.L., Buttazzoni L., Casadio R., Russo V. (2012). A genome wide association study for backfat thickness in Italian Large White pigs highlights new regions affecting fat deposition including neuronal genes. BMC Genom..

[11] Fan B., Onteru S.K., Du Z.-Q., Garrick D.J., Stalder K.J., Rothschild M.F. (2011). Genome-wide association study identifies loci for body composition and structural soundness traits in pigs. PLoS ONE.

[12] Zhang C., Bruce H., Yang T., Charagu P., Kemp R.A., Boddicker N., Miar Y., Wang Z., Plastow G. (2016). Genome wide association studies (GWAS) identify QTL on SSC2 and SSC17 affecting loin peak shear force in crossbred commercial pigs. PLoS ONE.

[13] Will K., Kuzinski J., Kalbe C., Palin M., Rehfeldt C. (2013). Effects of leptin and adiponectin on the growth of porcine myoblasts are associated with changes in p44/42 MAPK signaling. Domest. Anim. Endocrinol..

[14] Pérez-Enciso M., Clop A., Noguera J., Ovilo C., Coll A., Folch J., Babot D., Estany J., Oliver M., Diaz I. (2000). A QTL on pig chromosome 4 affects fatty acid metabolism: Evidence from an Iberian by Landrace intercross. J. Anim. Sci..

[15] Koskinen-Kolasa A., Vuolteenaho K., Moilanen T., Moilanen E. (2016). Adipokines leptin, adiponectin and resistin and their associations to MMPS, IL-6, COMP and radiographic severity of OA. Osteoarthr. Cartil..

[16] Takashima S., Nishii N., Kato A., Matsubara T., Shibata S., Kitagawa H. (2016). Molecular cloning of feline resistin and the expression of resistin, leptin, and adiponectin in the adipose tissue of normal and obese cats. J. Vet. Med. Sci..

[17] Schuster S.C. (2008). Next-generation sequencing transforms today’s biology. Nat. Methods.

[18] Spurlock M.E., Ranalletta M.A., Cornelius S.G., Frank G.R., Willis G.M., Ji S., Grant A.L., Bldwell C.A. (1998). Leptin expression in porcine adipose tissue is not increased by endotoxin but is reduced by growth hormone. J. Interferon Cytokine Res..

[19] Hood R., Allen C. (1977). Cellularity of porcine adipose tissue: Effects of growth and adiposity. J. Lipid Res..

[20] Kim K. (2002). Genetic Structure of Korean Native Pig Using Microsatellite Markers. Korean. J. Genet..

[21] Cho I.-C., Yoo C.-K., Lee J.-B., Jung E.-J., Han S.-H., Lee S.-S., Ko M.-S., Lim H.-T., Park H.-B. (2015). Genome-wide QTL analysis of meat quality-related traits in a large F 2 intercross between Landrace and Korean native pigs. Genet. Sel. Evol..

[22] Kim D., Seong P., Cho S., Kim J., Lee J., Jo C., Lim D. (2009). Fatty acid composition and meat quality traits of organically reared Korean native black pigs. Livest. Sci..

[23] White B., Lan Y., McKeith F., Novakofski J., Wheeler M., McLaren D. (1995). Growth and body composition of Meishan and Yorkshire barrows and gilts. J. Anim. Sci..

[24] Hubner N., Wallace C.A., Zimdahl H., Petretto E., Schulz H., Maciver F., Mueller M., Hummel O., Monti J., Zidek V. (2005). Integrated transcriptional profiling and linkage analysis for identification of genes underlying disease. Nat. Genet..

[25] Springer N.M., Stupar R.M. (2007). Allelic variation and heterosis in maize: How do two halves make more than a whole?. Genome Res..

[26] Bolger A.M., Lohse M., Usadel B. (2014). Trimmomatic: A flexible trimmer for Illumina sequence data. Bioinformatics.

[27] Kim D., Langmead B., Salzberg S.L.J.N.M. (2015). HISAT: A fast spliced aligner with low memory requirements. Nat. Methods.

[28] Liao Y., Smyth G.K., Shi W.J.B. (2014). featureCounts: An efficient general purpose program for assigning sequence reads to genomic features. Bioinformatics.

[29] Robinson M.D., Oshlack A.J.G.B. (2010). A scaling normalization method for differential expression analysis of RNA-seq data. Genome Biol..

[30] Robinson M.D., McCarthy D.J., Smyth G.K.J.B. (2010). edgeR: A Bioconductor package for differential expression analysis of digital gene expression data. Bioinformatics.

[31] Supek F., Bošnjak M., Škunca N., Šmuc T.J.P.O. (2011). REVIGO summarizes and visualizes long lists of gene ontology terms. PLoS ONE.

[32] Bindea G., Mlecnik B., Hackl H., Charoentong P., Tosolini M., Kirilovsky A., Fridman W.-H., Pagès F., Trajanoski Z., Galon J.J.B. (2009). ClueGO: A Cytoscape plug-in to decipher functionally grouped gene ontology and pathway annotation networks. Bioinformatics.

[33] Subramanian A., Tamayo P., Mootha V.K., Mukherjee S., Ebert B.L., Gillette M.A., Paulovich A., Pomeroy S.L., Golub T.R., Lander E.S. (2005). Gene set enrichment analysis: A knowledge-based approach for interpreting genome-wide expression profiles. Proc. Natl. Acad. Sci. USA.

[34] Smoot M.E., Ono K., Ruscheinski J., Wang P.-L., Ideker T.J.B. (2011). Cytoscape 2.8: New features for data integration and network visualization. Bioinformatics.

[35] Whittemore C.T. (1986). An approach to pig growth modeling. J. Anim. Sci..

[36] Kim S.-C., Jang H.-C., Lee S.-D., Jung H.-J., Park J.-C., Lee S.-H., Kim T.-H., Choi B.-H. (2014). Changes in expression of insulin signaling pathway genes by dietary fat source in growing-finishing pigs. J. Anim. Sci. Technol..

[37] Turman E.J., Andrews F. (1955). Some effects of purified anterior pituitary growth hormone on swine. J. Anim. Sci..

[38] Waltonand P.E., Etherton T.D. (1986). Stimulation of lipogenesis by insulin in swine adipose tissue: Antagonism by porcine growth hormone. J. Anim. Sci..

[39] Walton P.E., Etherton T.D., Evock C.M. (1986). Antagonism of insulin action in cultured pig adipose tissue by pituitary and recombinant porcine growth hormone: Potentiation by hydrocortisone. Endocrinology.

[40] Dunshea F., Harris D., Bauman D., Boyd R., Bell A. (1992). Effect of porcine somatotropin on in vivo glucose kinetics and lipogenesis in growing pigs. J. Anim. Sci..

[41] Pelleymounter M.A., Cullen M.J., Baker M.B., Hecht R., Winters D., Boone T., Collins F.J.S. (1995). Effects of the obese gene product on body weight regulation in ob/ob mice. Science.

[42] Chen X., Lin J., Hausman D.B., Martin R.J., Dean R.G., Hausman G.J. (2000). Alterations in fetal adipose tissue leptin expression correlate with the development of adipose tissue. Neonatology.

[43] Park S.-J., Ha J., Kim I.-S., Kwon S.G., Hwang J.H., Park D.H., Kang D.G., Kim T.W., Kim S.W., Kim C.W. (2015). Effects of LEP, GYS1, MYOD1, and MYF5 polymorphisms on pig economic traits. Ann. Anim. Sci..

[44] Barb C., Hausman G., Houseknecht K.J.D.A.E. (2001). Biology of leptin in the pig. Domest. Anim. Endocrinol..

[45] Sparrow J.C., Nowak K.J., Durling H.J., Beggs A.H., Wallgren-Pettersson C., Romero N., Nonaka I., Laing N.G. (2003). Muscle disease caused by mutations in the skeletal muscle alpha-actin gene (ACTA1). Neuromuscul. Disord..

[46] Nowak K.J., Wattanasirichaigoon D., Goebel H.H., Wilce M., Pelin K., Donner K., Jacob R.L., Hübner C., Oexle K., Anderson J.R. (1999). Mutations in the skeletal muscle α-actin gene in patients with actin myopathy and nemaline myopathy. Nat. Genet..

[47] Stratil A., Horák P., Nesvadbová M., Van Poucke M., Dvořáková V., Stupka R., Čítek J., Zadinová K., Peelman L.J., Knoll A. (2018). Genomic structure and expression of the porcine ACTC1 gene. Czech J. Anim. Sci..

[48] Kim D.-Y., Kim J.-M. (2021). Multi-omics integration strategies for animal epigenetic studies. Asian Australas. J. Anim. Sci..

