# Supplementary material for: Regulation of Swine Growth by Backfat Tissue during Growing and Finishing Stages
Source: Animals (Basel). 2021 Dec 9;11(12):3511. doi: 10.3390/ani11123511 (PMC8698142; doi:10.3390/ani11123511)
Supplement: Supplementary file 1 [file animals-11-03511-s001.zip › 1006_Backfat_supplementary figure.pdf]

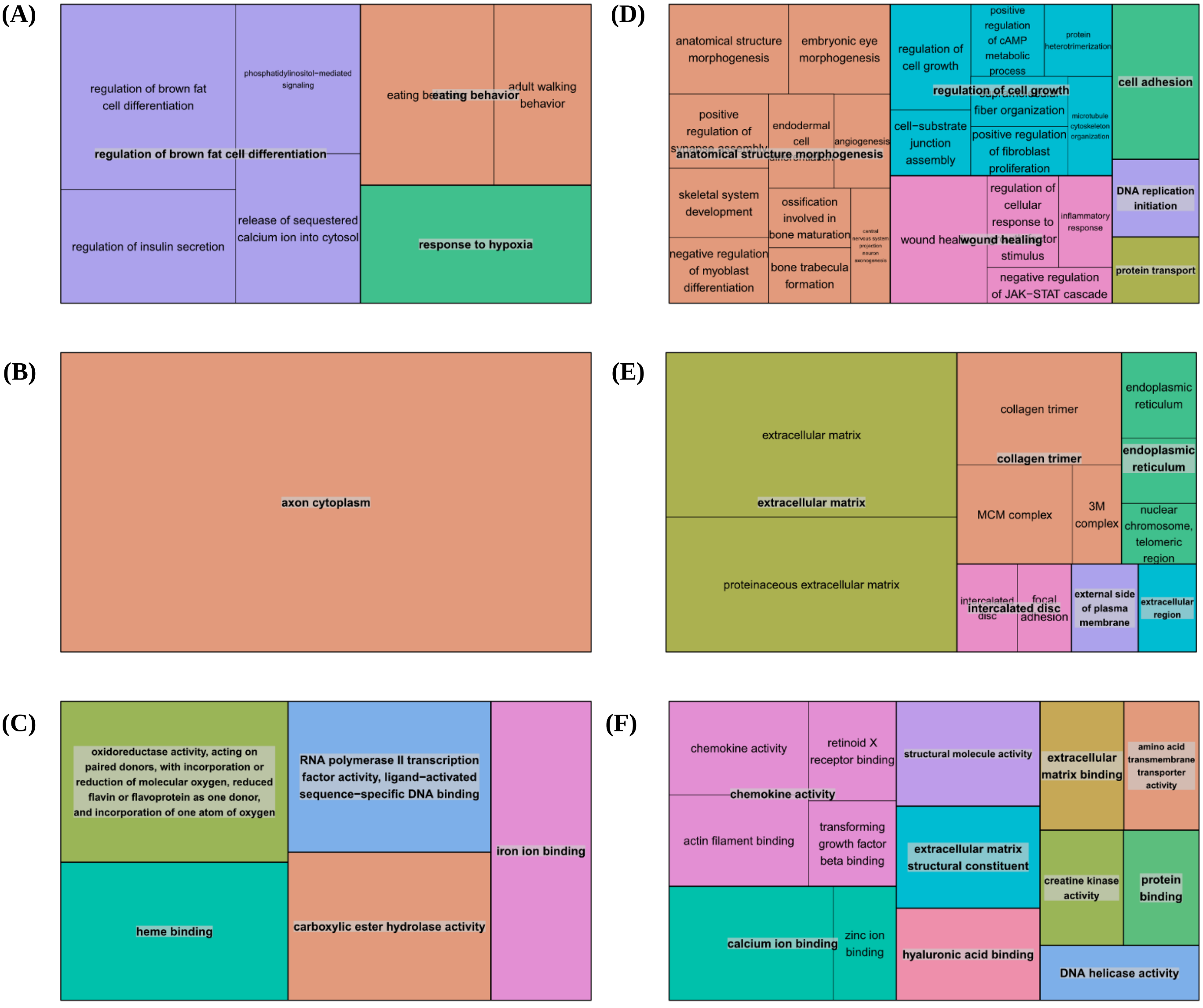

Figure S1. Revigo GO term analysis (A-C)Treemap view of Up regulated DEGs (A) Biological process category Treemap (B) Cellular Component category Treemap (C) Molecualr Function category Treemap (D-F)Treemap view of Down regulated DEGs (A) Biological process category Treemap (B) Cellular Component category Treemap (C) Molecualr Function category Treemap. Each rectangles size mean enrichment level. As size lager, enrichment level is higher. The lighter term name indicates representative term of groups.

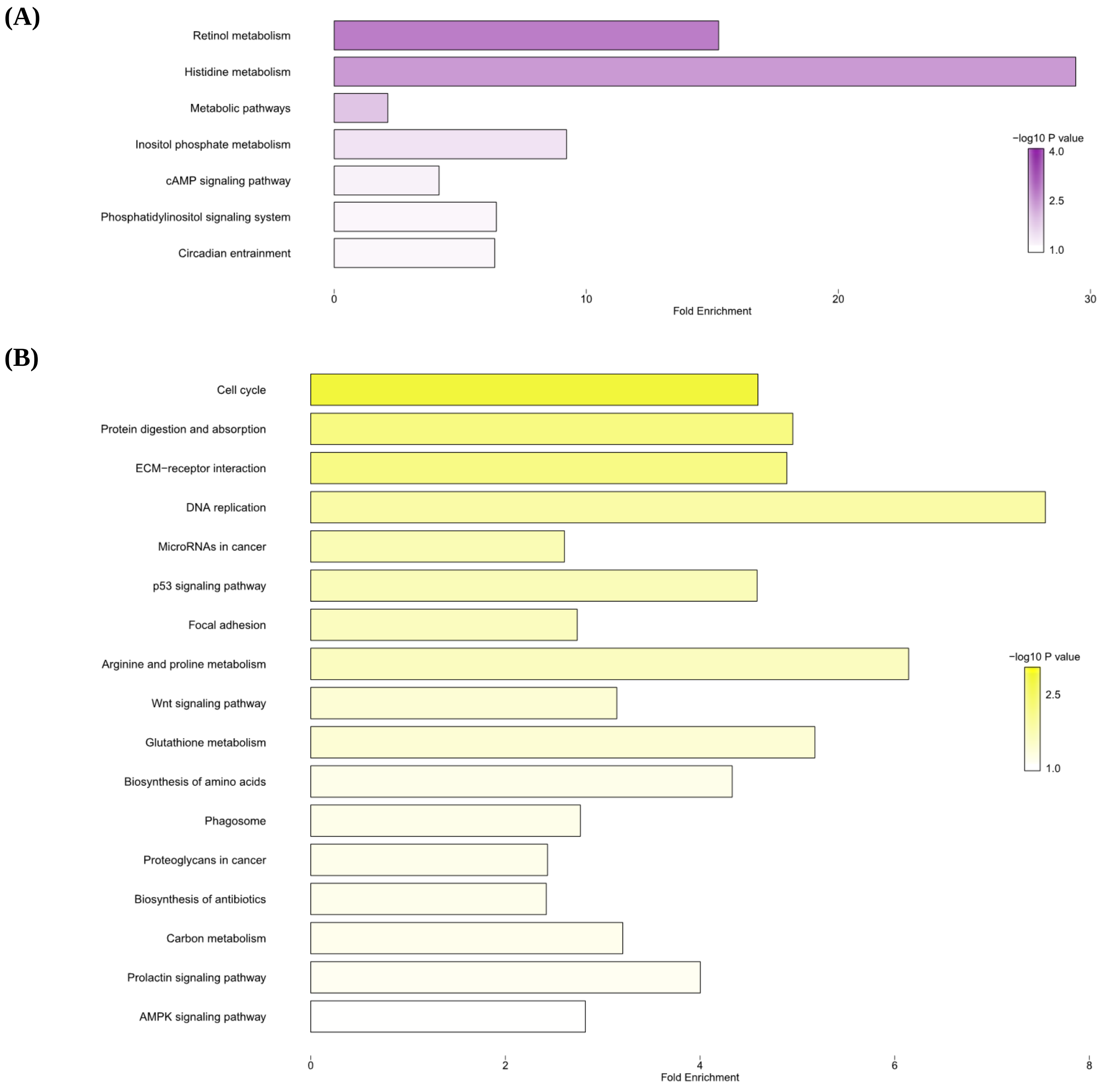

Figure S2. KEGG pathway graph (A) Up regulated DEGs KEGG pathway (B) Down regulated DEGs KEGG pathway. Each graph shows KEGG pathway of DEGs. First alignment threshold is $-\log_{10}$ Pvalue, and second alignment threshold is Fold enrichment.
